# Women’s Knowledge and Health Care-Seeking Behavior Regarding Pelvic Organ Prolapse in the West Bank, Palestine 

**DOI:** 10.1186/s12905-025-04023-4

**Published:** 2025-10-31

**Authors:** Doha Khaleel Moheasen, Ibtesam Medhat Mohamad Dwekat, Maha Sudki Nahal, Eman Awad Tayem

**Affiliations:** 1 Present Address: Department of Midwifery, Faculty of Health Professions, Al-Quds University, Jerusalem, Palestine; 2 Faculty of Nursing, Arab American University-Jenin, West Bank, Palestine

**Keywords:** Pelvic organ prolapse, Prevalence, Knowledge, health care-seeking behavior, West Bank

## Abstract

**Background:**

Pelvic organ prolapse is a prevalent condition affecting women of various ages; however, many remain unaware of this health issue and its potential complications. This study aimed to assess the knowledge, prevalence of reported POP symptoms, and healthcare-seeking behaviour among women in the West Bank.

**Methods:**

A cross-sectional study was conducted using a self-administered online questionnaire with a convenience sample of 317 women ages are 18 and older. Data were collected online via social media after contacting participants by phone and distributing a Google Form questionnaire. Data were analysed by using SPSS; Kruskal-Wallis and Mann-Whitney tests were also utilized.

**Results:**

Approximately 208 (65.6%) of the participants exhibited a low level of knowledge regarding Pelvic Organ Prolapse. The prevalence of reported pelvic organ prolapse symptoms was 182 (57.4%). Notably, 151 (82.9%) of the women who reported symptoms did not seek health care accordingly. Factors such as body mass index, residency, stress incontinence, chronic constipation, irregular menstruation, and menopause showed significant associations with the prevalence of reported symptoms of pelvic organ prolapse.

**Conclusion:**

Lower knowledge levels among Palestinian women were significantly associated with increased reports of POP symptoms, which correlated with limited health care-seeking behaviour. This issue is further complicated by cultural barriers to accessing care, political instability, limited availability of healthcare services, and varying levels of awareness among women.

**Implications:**

The findings highlight an urgent need for collaboration among healthcare providers, policymakers, and community representatives to address cultural barriers and ultimately improve access to healthcare by designing culturally sensitive health promotion campaigns targeting women aged 30 and above.

**Supplementary Information:**

The online version contains supplementary material available at 10.1186/s12905-025-04023-4.

## Introduction

Pelvic Organ Prolapse (POP) refers to the descent and herniation of one or more of the anterior and posterior vaginal walls, the uterus, cervix, or the apex of the vagina [1]. It is among the most common gynecologic health issues affecting women, particularly those who are postmenopausal or have conditions that elevate intra-abdominal pressure, such as chronic coughing or obesity [2]. Approximately 50% of women are expected to develop POP at some point in their lives [3]. The current global prevalence of POP is approximately 30.9% [4]. Various factors are associated with the prevalence of POP, including educational status, socioeconomic conditions, and menopausal status [5]. Significant risk factors also include multiparty, obesity (defined as a body mass index (BMI) greater than 25 kg/m²), and participation in heavy physical activity [6, 7]. Furthermore, the history of constipation, chronic respiratory diseases, and birthing babies with high birth weights are recognized as additional risk factors for POP [8]. Common symptoms of POP include vaginal or pelvic pressure, urinary and bowel incontinence, sexual discomfort, and dyspareunia. These symptoms can significantly affect the daily activities of women experiencing them and diminish their overall quality of life. Additionally, the health-seeking behavior of women with POP is often shaped by their socioeconomic status, the limited understanding of the condition and its symptoms and the treatment options that are available to them. Despite ongoing efforts to improve access to diagnosis and treatment services, many women with POP remain untreated for years, often delaying care until the condition has advanced significantly [9]. Factors such as low socioeconomic status, cultural and spiritual beliefs, feelings of shame, and fear of stigma have a major impact on health-seeking behavior. Therefore, it is essential to raise awareness among women about POP, its symptoms, and potential complications to promote early detection. Increased knowledge will facilitate effective management, help prevent complications, and reduce healthcare costs for both institutions and individuals [10].

In the Arab world, the prevalence of POP among women in Arab countries varies significantly, with reported rates of 29.6% among Emirati women [8], 67.7% among Saudi women [11], and 49.8% among Lebanese women [12]. This disparity in prevalence is influenced by various factors, including cultural and socioeconomic conditions. Additionally, women’s healthcare behavior, attitudes toward childbirth, practices such as parity [8], economic factors [13], and health education [14] all contribute to the higher incidence of POP in the Arab region.

### Palestinian context

Currently, no studies have identified the prevalence rate of POP in Palestine, and there is a scarcity of research on this topic. However, a few studies have examined the prevalence of stress urinary incontinence (SUI) among Palestinian women and its impact on their quality of Life. One study indicated that 26.9% of participants reported experiencing symptoms of SUI [15]. There are specific healthcare barriers in Palestine that exacerbate issues related to POP compared to other countries. The Palestinian community is conservative, making it taboo for women to address pelvic health issues openly due to sociocultural and normative constraints. Many women view POP as a shameful and embarrassing condition, leading them to conceal their symptoms and avoid discussing them. As a result, precisely identifying the prevalence of POP among Palestinian women remains challenging. Additionally, political instability, ongoing war in Gaza, poor economic conditions in West Bank, poverty, and the limited access to healthcare complicate the situation. These factors often lead women to prioritize other issues over their health, causing them to adapt to their symptoms instead. Furthermore, a lack of knowledge about the symptoms, diagnosis, and treatment of POP contributes to women’s reluctance to seek healthcare, worsening their suffering. This study addresses a critical research gap in the Palestinian context by evaluating the prevalence of reported POP symptoms, women’s knowledge about POP, and their healthcare-seeking behaviors.

The Health Belief Model (HBM) is one of the most commonly used psychological frameworks for explaining and predicting health-related behaviors. The model is useful especially in the context of disease prevention and the development of health-related interventions. The six core constructs of HBM are perceived susceptibility, perceived severity, perceived benefits, perceived barriers, self-efficacy, and cues to action [16]. Numerous studies have utilized the HBM framework to elucidate their findings and to create educational health interventions [17, 18]. This study aimed to assess the knowledge, prevalence of reported POP symptoms, and healthcare-seeking behavior among women in the West Bank, Palestine.

## Materials and methods

A cross-sectional study utilizing a self-administered online questionnaire was conducted in the southern region of the West Bank, specifically in Bethlehem and Hebron, Palestine, from November 2023 to August 2024. The study included women aged 18 and above residing in the Hebron and Bethlehem governorates. Exclusions applied to midwives, doctors, and pregnant women. Additionally, women from areas lacking internet access and those with lower digital Literacy were also excluded. The sample size was determined by using a single population proportion formula, with a 95% confidence level, a significance level of 0.05, and an expected prevalence of 30.9%, reflecting the global prevalence of pelvic organ prolapse (POP) [4]. Consequently, the calculated sample size was 329 women. A proportional quota sampling method was employed to ascertain the number of women to be selected from each governorate, based on the total population of each area. The quotas established were 220 women from Hebron and 110 from Bethlehem. A convenience sampling method was then used to achieve the required sample size of women from each governorate.

### Study instrument

A self-administered online questionnaire was used to collect data from the participating women. It was developed by the researchers to assess women’s knowledge about POP, apparent symptoms, and factors influencing health care-seeking behavior in the southern area of the West Bank, Palestine. The final questionnaire included four domains: sociodemographic and other characteristics, knowledge of POP, symptoms of POP, and factors affecting health care-seeking behavior. The questionnaire was based on a comprehensive literature review [8, 12, 19, 20]. The sociodemographic, menstrual, obstetric, and medical history section contained 26 items. The knowledge scale regarding POP included 17 items; 11 items were adopted from the Arabic version of the Prolapse and Incontinence Knowledge Questionnaire (PIKQ) [21], while 6 items were added which based on expert input during the validation process. Knowledge was assessed using a three-point Likert scale (Agree, Disagree, I don’t know). Previous studies, using the Pelvic Floor Disability Index (PFDI-20 [22, 23]), identified six important symptoms of POP. The studied symptoms were evaluated using a four-point Likert scale, which included the options of “always,” “most of the time,” “sometimes,” and “never.” To assess barriers to health care-seeking behavior among women who reported noticeable symptoms, the study included 10 items measured on a two-point Likert scale (yes or no). Respondents had the option to select multiple barriers for not seeking care. The items were adapted from prior research [6, 24–26] and incorporated recommendations from experts, along with feedback from women who reviewed the questionnaire during its development and validation process. Subsequently, the researchers reviewed and modified the entire questionnaire to ensure it was appropriate for women in Palestinian cultures. It was then translated into Arabic and proofread by two experts fluent in both Arabic and English. For more details, 1 & 2: the Arabic and English versions of the questionnaire were provided in supplementary files 1and 2.

According to the validation process, content and face validity were conducted to prove the clarity, understandability, relevancy, and adaptability of the questionnaire to the Palestinian context. The Arabic version of the questionnaire was sent to six experts for content validity. The group of experts included two midwives, one reproductive health officer, and three faculty members from the Nursing and Midwifery Department at Al-Quds and Bethlehem Universities. After agreeing to participate in the validation process, the experts received an email containing the questionnaire along with the study objectives. They were asked to review the domains and their items and to provide feedback on each item if possible. 

One expert suggested adding more items to the knowledge section, leading to additional items being incorporated into the Prolapse and Incontinence Knowledge Questionnaire (PIKQ). The items were subsequently organized into three sections: risk factors, diagnosis, management and treatment of POP. Another expert requested clarification of certain items, such as the term “hard work.” The feedback from all experts was valuable, enhancing the content and clarity of the questionnaire items, and it was all considered to refine the domains and their items.

Face validation was conducted with 10 participants to ensure the items in the questionnaire were understandable and clear. Women were encouraged to provide verbal or written feedback, and all reported that the items were clear and easy to understand. Additionally, pilot testing and reliability assessment of the questionnaire domains were performed with 30 women. This process evaluated recruitment procedures, the time required to complete the questionnaire, any technical issues, and overall difficulty with the questionnaire format. The women provided positive feedback. Furthermore, the reliability of the questionnaire was confirmed by calculating Cronbach’s alpha for each domain: knowledge (α = 0.933), symptoms (α = 0.702), and barriers to healthcare- seeking behavior (α = 0.848), indicating that the questionnaire is both valid and reliable.

### Data collection

Data were collected over two months, using a self-administered online questionnaire, which ran from November 2023 to January 2024. Approximately 317 completed questionnaires were returned to the researcher via email, resulting in a response rate of 96%. The online data collection process was conducted by the main researcher with the assistance of two data collectors; one located in Hebron and the other in Bethlehem. These data collectors communicated with the women through social media platforms, including large WhatsApp and Facebook groups. Women who met the inclusion criteria and agreed to participate in the study received a Link to the questionnaire via private messages. Participation was voluntary, and informed consent was obtained from each participant prior to completing the online questionnaire. The purpose of the study and the inclusion criteria were clearly explained to participants before sending them the questionnaire. Additionally, a consent form was included within the online questionnaire itself. The self-administered online questionnaire took approximately 10 minutes to be completed. The online data collection method was used due to the main researcher’s inability to reach each woman individually, stemming from closures and transportation difficulties. The risk of movement between towns was heightened by the ongoing Israeli war on Gaza and the political instability in the West Bank. Ethical approval for the study was granted by the Ethical Committee at Al-Quds University, in accordance with the Declaration of Helsinki standards, with reference number 285/REC/2023.

### Statistical analysis

The analysis of 317 completed questionnaires was conducted using the Statistical Package for Social Sciences (SPSS) version 25. Variables were defined and coded, and the data were checked for missing values. Due to the non-normal distribution of the data, nonparametric tests, including the Mann-Whitney and Kruskal-Wallis tests, were employed. Also, the Shapiro-Wilk test was done, showing that the data does not follow a normal distribution as expected. See Table 1.

**Table 1:** Shapiro-Wilk and Kolmogorov-Smirnov tests results

Variable	Kolmogorov-Smirnov	*P*-Value	Shapiro-Wilk	*P*-Value
Age	0.23	**0.000**	0.80	**0.000**
BMI	0.22	**0.000**	0.84	**0.000**
Educational level	0.34	**0.000**	0.75	**0.000**
Education related to medical science	0.27	**0.000**	0.78	**0.000**
Occupation	0.39	**0.000**	0.68	**0.000**
Residency	0.24	**0.000**	0.81	**0.000**
Marital status	0.54	**0.000**	0.15	**0.000**
monthly income	0.35	**0.000**	0.64	**0.000**

Descriptive statistics were utilized to outline the sociodemographic characteristics, menstrual and obstetrical history, and gynecologic and medical history of the participants. The level of knowledge regarding POP was assessed by calculating frequencies and percentages. The criteria for categorizing how cutoff scores for “low” and “high” knowledge were determined based on a previous study [14]. Participants who answered questions correctly received two points and were classified as having “high” knowledge. Conversely, participants who chose “I don’t know” or provided incorrect answers received one point and were classified as having “low” knowledge. The Kruskal-Wallis Test was employed to examine the relationships between knowledge levels and sociodemographic characteristics. Additionally, this test was used to investigate the relationships between the prevalence of reported POP symptoms and both demographic characteristics and obstetric history. The Mann-Whitney Test was applied to analyze the relationship between knowledge levels and monthly family income, as well as current POP symptoms. It was also used to explore the relationships between POP symptoms and both monthly family income and medical menstrual history.

The prevalence of reported POP symptoms was calculated using percentages and frequency. Responses were coded such that participants who answered “never” received one point, while those who answered “sometimes,” “often,” or “always” received two points. Thus, participants with a score of 1 were classified as not having any symptoms, while those with a score of 2 were classified as having symptoms.

## Results

### Sociodemographic characteristics of the participants

A total of 317 women responded to the online questionnaire across the two governorates of Hebron and Bethlehem. Approximately 125 (39.4%) of the participating women ages range from 30 to 40 years, while 112 (35.3%) were 40 years old or above. Around 135 (42.6%) of the participants were classified as overweight. Nearly half of the respondents, 153 (48.2%), resided in villages. The majority, 196 (61.8%), had completed their university education, and 159 (81.12%) of those degrees were not related to the medical field. The medical field includes professions like doctors, midwives, and nurses. See Table 2.

**Table 2:** The sociodemographic characteristics of the participants (*n* = 317)

Variables		*N*	(%)
Age			
18–29		80	25.2
30–40		125	39.4
above 40		112	35.3
Body Mass Index:			
Underweight		6	1.9
Normal		81	25.6
Overweight		135	42.6
Obese		93	29.3
Residency:			
City		108	34.1
Village		153	48.2
Camp		56	17.7
Educational level			
Basic education		16	5
High school		94	29.7
College or university		207	65.3
University major related to medical science			
Yes		37	18.9
No		159	81.1
Occupation:			
No formal occupation		198	62.5
Office job		95	30
Jobs require strenuous physical activities		24	7.6
Monthly family income:			
Less than 3500 ILS		151	47.6
More than 3500 ILS		166	52.4

*ILS *Israeli new shekel

### The menstrual and obstetric history of the participants

The majority of participants, 238 (75.1%), reported they have regular menstrual cycles. Additionally, 286 (90.2%) had not yet reached menopause. More than half of the participants, 183 women (57.7%), had four or more births. Nearly half of the participants, 144 (45.4%), had four or more normal vaginal deliveries (NVD). Among the participants, 40 (12.6%) had one to three deliveries via instrumental methods, such as vacuum or forceps. See Table 3.

**Table 3:** The menstrual and obstetric history of the participants (*n* = 317)

Variables	*N*	(%)
Menstrual history		
Regular	238	75.1
Irregular	79	24.9
*Reach menopause		
Yes	31	9.8
No	286	90.2
Obstetrical history		
** Parity		
Nullipara	10	3.2
Para 1	23	7.3
Para 2–3	101	31.9
Para 4 and above	183	57.7
Number of vaginal births		
1	28 (8.8)	8.8
2–3	101	31.9
4 and above	144	45.4
Number of C/S births		
1	66	20.8
2–3	30	9.5
4 and above	12	3.8
1	35	11
2–3	5	1.6
Births weighing over 4 kg		
Yes	53	16.7
No	254	80.1

* Menopause: The stage in a woman's Life when her ovaries stop producing hormones and menstrual periods cease, defined as the absence of menstruation for 12 consecutive months

** Parity: The total number of pregnancies that have reached the threshold of viability

### Gynecologic and medical history of the participants

Approximately 118 participants (37.2%) reported experiencing stress incontinence. Additionally, 6 participants (1.9%) had undergone surgery for the treatment of POP. Regarding the medical history of the participants, 33 women (10.4%) had a chronic chest illness and 62 (19.6%) of women reported a history of chronic constipation. See Table 4.

**Table 4:** The gynaecological and medical history of the participants (*n* = 317)

Variables	*N*	(%)
*** Stress incontinence		
Yes	118	37.2
No	199	62.8
Surgery for stress incontinence treatment		
Yes	6	1.9
No	311	98.1
Previous surgery for POP		
Yes	6	1.9
No	311	98.1
Chronic chest illness		
Yes	33	10.4
No	284	89.6
Constipation		
Yes	62	19.6
No	255	80.4
Diabetes		
Yes	23	7.3
No	294	92.7
Smoking cigarettes or shisha		
Yes	32	10.1
No	285	89.9
Diagnosed of pelvic cancer		
Yes	1	0.3
No	316	99.7

***Stress incontinence: occurs when urine leaks as pressure is put on the bladder, such as during exercise, coughing, sneezing, laughing, or lifting heavy objects

### Participants’ knowledge level and information sources regarding POP

Approximately 95 (30%) of the participants reported that their primary source of information about POP was the surrounding community. Moreover, around 208 (65.6%) of the participants exhibited a low level of knowledge, while 109 (34.4%) participants demonstrated a high level of knowledge. Specifically, 121 women (38.2%) had a low level of knowledge regarding risk factors, 120 (37.9%) were unaware of symptoms, and 126 (39.7%) lacked understanding about the treatment options of POP. Table 5 for more details.

**Table 5:** Level of participants’ knowledge regarding POP (*n* = 317)

Variables	High knowledge *N* (%)	Low knowledge *N* (%)
Knowledge regarding risk factors	121 (38.2)	196 (61.8)
knowledge regarding diagnosing symptoms	120 (37.9)	197 (62.1)
knowledge regarding treatment	126 (39.7)	191 (60.3)
Total level of knowledge	109 (34.4)	208 (65.6)

### Factors associated with participants’ knowledge level about POP

There are two factors linked to the level of knowledge about POP; the field of medical education and the presence of symptoms. Participants who were enrolled in a medical-related field demonstrated a low level of knowledge regarding POP (95% CI: 0.97, 1.076, P value = 0.00). Furthermore, women with low knowledge about POP reported a higher incidence of symptoms (95% CI: 1.367, 1.54, P value = 0.001). Conversely, no significant relationship was identified between knowledge of POP and the sociodemographic characteristics of the women. See Table 6.

**Table 6:** Factors associated with the participants’ knowledge level about POP

Variables		*N* (%)	H test	U test	CI	Mean	*P*-value
Age	18–29 years	80 (25)	1.66				0.436
	30–40 years	125 (39.4)					
	40 years & above	112 (35.3)					
Residence	City	108 (34)	1.78				0.409
	Village	151 (47.6)					
	Camp	56 (17.6)					
Level of education	Basic education	16 (5)	1.93				0.587
	High school	94 (29.7)					
	College or University	207 (65.3)					
Occupation	Housewives	198 (62.4)	3.6				0.165
	Office job	95 (29.9)					
	Jobs involving strenuous activity	24 (7.5)					
Medical- related field	Yes	40 (12.6)	20.6	2039.5	(0.98, 1.08)	1.03	**< 0.001**
	No	159 (50.1)		1479.0	(1.31, 1.46)	1.38	0.805
	University education not completed	118 (37.2)			(1.31, 1.49)	1.40	
Presence of symptoms	No	135 (42.5)		9974.0	(1.37, 1.54)	1.45	**< 0.001**
	Yes	182 (57.4)			(1.12, 1.33)	1.26	

*H *Kruskal-Wallis Test*, U *Mann-Whitney*, CI *Confidence interval

*Medical-related field: all medical fields except midwives and doctors

### The Prevalence of Reported POP Symptoms

The prevalence of reported POP among the participants was 182 (57.4%). The most frequently reported symptom was lower abdominal pressure, experienced by 139 participants (43.8%). The next most common symptoms included a feeling of heaviness in the pelvic area that was reported by 113 participants (35.7%), and a sensation of incomplete bladder emptying, noted by 112 participants (35.3%). See Table 7.

**Table 7:** The prevalence of reported POP symptoms (*n* = 317)

Symptom	Yes *N* (%)	No *N* (%)
Lower abdominal pressure	139 (43.8)	178 (56.2)
Heaviness in the pelvic area	113 (35.7)	204 (64.3)
Visible or palpable bulge in or outside the vaginal area	97 (24.9)	238 (75.1)
The need to insert a finger into the vagina to urinate completely	29 (9.2)	288 (90.9)
A sensation of incomplete bladder emptying	112 (35.3)	205 (64.7)
The need to apply pressure to the vagina or rectum to facilitate defecation	95 (29.96)	222 (70.0)
Total prevalence os Symptoms	(182) 57.4	

### Factors associated with the prevalence of reported POP symptoms

Six variables were found related to the prevalence of reported POP symptoms: the participants’ residence, body mass index (BMI), stress incontinence, constipation, menstrual history, and menopause status. Regarding residence, participants living in camps demonstrated a significant relationship with the prevalence of reported POP symptoms (95% CI: 1.40, 1.59, p-value = 0.021). In terms of BMI, overweight or obese women were more likely to experience POP symptoms (95% CI: 1.47, 1.64, p-value = 0.045), as illustrated in Table 8.

Participants who experienced stress incontinence were also more likely to report POP symptoms (95% CI: 1.67, 1.83, p-value 0.001). Furthermore, individuals with chronic constipation had a significant association with reported POP symptoms (95% CI: 1.79, 1.91, p-value 0.001). Participants with irregular menstrual cycles and menopausal women were more likely to report POP symptoms, as shown in Table 9. Conversely, there was no significant association between the prevalence of reported POP symptoms and demographic or obstetric history and previous medical history (p-value > 0.05).

**Table 8:** Association between reported POP symptoms and sociodemographic and obstetric factors (*n* = 317)

Variables		No (%)	H	CI	Mean	*P*-Value
Age	18–29 years	80 (25)	0.70			0.703
	30–40 years	125 (39.4)				
	40 years and above	112 (35.3)				
Occupation	Housewives	198 (62.4)	0.50			0.782
	Office job	95 (29.9)				
	Jobs involving strenuous activity	24 (7.5)				
Residency	City	108 (34.1)	7.70	(1.39, 1.59)	1.49	**0.021**
	Village	15348.1		(1.51, 1.67)	1.59	
	Camp	56 (17.7)		(1.59, 1.83)	1.71	
Family monthly income	Less than 3500 ILS	151 (47.6)				0.328
	More than 3500 ILS	166 (52.3)				
Body mass index (BMI)	Underweight	6 (1.8)	8.06	(0.79, 1.88)	1.33	**0.045**
	Normal	81(25.5)		(1.39, 1.62)	1.51	
	Overweight	135 (42.5)		(1.47, 1.64)	1.56	
	Obese	93 (39.3)		(1.59, 1.78)	1.69	
Parity	Nullipara	10 (3.15)	2.02			0.508
	para 1	23 (7.2)				
	para 2–3	101 (31.8)				
	para 4 and above	183 (57.7)				

*H* Kruskal-Wallis Test

**Table 9:** Association between reported POP symptoms and medical/gynecologic history (*n* = 317)

Variables		*N* (%)	U test	CI	Mean	*P*- Value
Stress incontinence	Yes	118 (37.2)	8531	(1.67, 1.83)	1.75	**< 0.001**
	No	199 (62.7)		(1.40, 1.54)	1.47	
Surgery for stress incontinence treatment	Yes	6 (1.8)	686.5			0.169
	No	311 (98.1)				
Previous surgery for POP treatment	Yes	6 (1.8)	686.5			0.169
	No	311 (98.1)				
Chronic chest illness	Yes	33 (10.4)	4043.5			0.132
	No	284 (89.5)				
Constipation	Yes	62 (19.5)	4988	(1.79, 1.91)	1.87	**< 0.001**
	No	255 (80.4)		(1.44, 1.56)	1.50	
Diabetes	Yes	23 (7.2)	3255.0			0.728
	No	294 (92.7)				
Smoking Cigarettes or Shisha	Yes	32 (10)	3985.0			0.172
	No	285 (89.9)				
Menstrual history	Regular cycle	238 (75)	7872.5	(1.47, 1.60)	1.53	**0.011**
	Irregular cycle	79 (24.9)		(1.59, 1.80)	1.70	
Reach Menopause	Yes	31 (9.7)	3608.5	(1.58, 1.90)	1.74	**0.047**
	No	286 (91.1)		(1.50, 1.61)	1.56	

*U* Mann-Whitney

#### Health care-seeking behavior (n = 182)

Around 151 (82.96%) of the participants who reported POP symptoms had not sought health care. Only 31 (17.03%) sought medical attention. Several barriers keep women with POP symptoms from getting treatment and the participants had the option to select multiple barriers for not seeking care. The most commonly reported barrier was “the symptoms are not annoying at first,” with approximately 106 (70.19%) of participants agreeing with this statement. Additionally, 103 (68.2%) women did not seek health care because they had believed their condition was normal. Other reasons for not seeking health care included feelings of embarrassment when discussing the issue with a doctor, the inability to afford medical treatment, concerns about the degree to which treatment might affect future pregnancies, and societal or cultural factors. Table 10 outlines the barriers faced by women with POP symptoms when attempting to seek care.

**Table 10:** Barriers to health Care-Seeking behavior among women with POP symptoms (*n* = 151)

Variables	Yes (%)	No (%)
The symptoms are not annoying at first.	106 (70.19)	45 (29.8)
Belief that the condition they suffer from is normal.	103 (68.2)	48 (31.78)
Misunderstood that their problem is POP.	91 (60.26)	60 (39.73)
Lack of knowledge of the existence of a medical treatment for the problem	73 (48.3)	78 (51.65)
Embarrassment related to the medical assessment that might be conducted	71 (47.01)	80 (52.98)
Fear that treatment will result solely in surgery	64 (42.38)	87 (57.6)
Feeling embarrassed to discuss POP with the doctor	58 (38.4)	93 (61.58)
Inability to pay the cost of medical treatment.	44 (29.1)	107 (70.86)
Concerns about the potential impact on future pregnancies.	40 (26.49)	111 (73.5)
Societal-cultural reasons.	36 (23.84)	115 (76.1)

## Discussion

### The level of knowledge about POP

The study aimed to assess women’s knowledge of POP, the prevalence of reported symptoms, and health-seeking behaviors among women in West Bank, Palestine. The limited awareness among Palestinian women regarding POP may stem from the sensitivity surrounding this issue, it is often viewed as an embarrassing and private topic, particularly in a conservative society the Palestinian community. These findings align with previous research indicating a lack of knowledge and awareness about prolapse among women [27–31]. A notable finding of this study suggests a potential link between limited awareness of POP and an increased risk of developing the condition. Additionally, routine screening for women’s health and enhancing awareness of POP symptoms are recommended [28, 30]. In this context, knowledge serves as a form of empowerment and a potential preventive measure against diseases. Lack of knowledge about POP treatment may delay the pursuit of care and contribute to a deterioration in health conditions [9, 32].

### The prevalence of reported POP symptoms and its associated factors

The prevalence of reported POP symptoms in the West Bank remains in correspondence with the international prevalence [12] and the Eastern Mediterranean region [33]. Several pieces of evidence suggest that the prevalence of POP in Palestine is likely comparable to that in other Eastern Mediterranean regions, which face similar risk factors and women’s health issues [13, 14, 33]. In contrast, the prevalence of POP in South Asian countries, such as China and Pakistan, is lower, reported at 9.10% [34] and 10.3% [35], respectively. This indicates that the prevalence in Palestine remains high.

The variation in the prevalence of POP among different countries may be influenced by several factors; it includes demographic and obstetric characteristics, economic and sociocultural issues, healthcare systems, community dynamics, levels of awareness, healthcare-seeking behavior, lifestyle choices, smoking, obesity, and the methodologies used to calculate prevalence rates. Understanding these factors is crucial for comprehending the disparities in POP prevalence across various countries. Additionally, it is important to note that most previous studies on POP prevalence have relied on symptom-based diagnoses. This approach may overlook asymptomatic cases of POP and might not accurately represent the true prevalence of the condition.

Multiple factors associated with the prevalence of POP were identified in this study, consistent with findings from previous research. These factors include being overweight or obese [8, 12, 16, 17, 36], experiencing constipation [8, 12], and having stress incontinence [37]. Additionally, women with irregular menstrual cycles and those who are menopausal [38] also reported symptoms of POP. This finding contradicts another study [39] that concluded there was no statistical association between postmenopausal status and pelvic organ prolapse (POP).

### Barriers to health care-seeking behavior and health belief model (HBM)

The majority of participants with symptoms of POP did not seek appropriate care, which could harm their health and also affect their quality of life. A previous study [12] also reported similar findings. POP often impacts women’s sexual, social, psychological, and physical functioning, affecting their daily activities and reducing productivity [35]. The influence of sociodemographic and cultural barriers was evident in the participants’ responses regarding the obstacles they perceived in accessing healthcare.

In this study, sociodemographic and cultural barriers, particularly the sensitivity of the condition and embarrassment about seeking care, may significantly explain the participants’ responses to perceived obstacles to health care-seeking behavior. These findings align with the principles of the Health Belief Model (HBM), which offers a useful framework for understanding the psychological factors that influence individuals to seek or avoid preventive health services or treatment [16]. Although this study did not explicitly evaluate all six-core constructs of HBM, which include perceived susceptibility, perceived severity, perceived benefits, perceived barriers, self-efficacy, and cues to action [16], it did reveal significant gaps in awareness that may have influenced health care-seeking behavior. Palestinian women in this study lacked recognition of their vulnerability to POP and underestimated the seriousness of their symptoms, often normalizing them as a typical part of womanhood. This limited awareness likely contributed to delays or avoidance in seeking appropriate care. These results illustrate the importance of culturally sensitive outreach programs aligned with the HBM. Interventions should raise awareness about the susceptibility and severity of POP using real-life stories and peer education while addressing women’s embarrassment through community dialogue, involvement of cultural leaders, and training female healthcare providers. Strengthening self-efficacy through education, support groups, and counselling can empower women to recognize the benefit of seeking early care. Healthcare providers, policymakers, and community representatives should intensify their efforts to raise awareness among women, diminish stigma, and address barriers to seeking care. This initiative is critical for educating women about POP. Ultimately, it can lead to improved access to medical services and encourage women to visit gynecologic clinics at the first sign of symptoms for early detection and management, thus promoting the well-being of Palestinian women affected by POP. Additionally, tackling cultural barriers and stigma is vital for both women and the community, as it encourages them to express their challenges and cultivates a greater willingness to seek care.

### Strength and limitations

One of the strengths of this study is a pioneering one in West Bank to assess the prevalence of reported symptoms of POP, health care-seeking behavior, and the knowledge of Palestinian women on this topic. The results provide baseline data for other Palestinian researchers regarding POP, which can be useful for conducting interventional studies aimed at raising awareness among women about POP symptoms, risk factors, and prevention methods.

This study has several limitations, including its focus on only one region in the southern West Bank, which excludes the central and northern areas. Furthermore, due to political issues and safety concerns in the West Bank, as well as the ongoing war in Gaza, data collection was conducted online. This method may introduce selection bias, as it primarily reaches women who use the internet while excluding those who are less familiar with technology. Consequently, this exclusion may lead to an underrepresentation of vulnerable groups, which could affect the study’s results, especially concerning the reported prevalence of POP symptoms.

Additionally, the reliance on self-reported information may introduce response bias, resulting from inaccurate recall or a tendency to provide socially desirable answers. 

Moreover, relying on self-reported data, especially concerning symptoms, could result in either an overestimation or underestimation of the actual prevalence of conditions like POP. These limitations hinder the dissemination of the study’s findings. Therefore, future research that relies on clinical diagnoses would yield a more robust and accurate estimate of the POP prevalence, resulting in more precise and reliable findings. In fact, the interplay of selection bias and self-reporting bias may compromise the validity of the findings, especially concerning the reported prevalence of POP symptoms. Subsequent studies should include a larger sample size and utilize a combination of data collection methods, such as both self-reported and clinical assessment-based approaches, to ensure validity and generalizability issues.

## Conclusion

Lower knowledge levels among Palestinian women were significantly associated with increased reports of POP symptoms, which correlated with limited health care-seeking behaviour. This situation creates a cycle of suffering and silence for many women. Cultural barriers to accessing care, political instability, limited availability of healthcare services, and varying levels of awareness among women further complicate these issues.

Additionally, participants who were overweight or obese, lived in camps, suffered from constipation, experienced stress incontinence, had irregular menstrual cycles, or were menopausal showed a higher prevalence of POP symptoms. It is important for women to modify their lifestyles by making dietary changes, engaging in regular exercise, losing weight, and managing existing medical conditions. Therefore, addressing cultural barriers, enhancing education, and improving access to care are crucial steps in promoting the well-being of Palestinian women dealing with POP. Urgent collaboration among healthcare providers, policymakers, and community leaders is necessary to design culturally sensitive health promotion campaigns targeting women aged 30 and above. Since the findings of the study are based on reported symptoms, future research should include clinical assessments to ensure accurate evaluations of women experiencing these symptoms and to improve the calculation of POP prevalence.

## Supplementary Information

Supplementary Material 1.

Supplementary Material 2.

## Data Availability

The datasets supporting the conclusion of this article are available.

## Abbreviations

POP: Pelvic Organ Prolapse
PFD: Pelvic Floor Disorder
PIKQ: Prolapse and Incontinence Knowledge Questionnaire
SUI: Stress Urinary Incontinence
PFDI: Pelvic Floor Disability Index
BMI: Body Mass Index
NVD: Normal Vaginal Delivery
C/S: Caesarean Section
WHO: World Health Organization
SPSS: Statistical Package of Social Science
U: Mann-Whitney
H: Kruskal– Wallis
CI: Confidence Interval
HBM: Health Belief Model
