# Correction: Women’s knowledge and health care-seeking behavior regarding pelvic organ prolapse in the West Bank, Palestine

**DOI:** 10.1186/s12905-025-04243-8

**Published:** 2026-01-12

**Authors:** Doha Khaleel Moheasen, Ibtesam Medhat Mohamad Dwekat, Maha Sudki Nahal, Eman Awad Tayem

**Affiliations:** 1 Present address: Department of Midwifery, Faculty of Health Professions, Al-Quds University, Jerusalem, Palestine; 2 Faculty of Nursing, Arab American University-Jenin, West Bank, Palestine

**Correction: Moheasen et al. BMC Women’s Health 25, 529 (2025)**

**https://doi.org/10.1186/s12905-025-04023-4**

In this article [1], the title was incorrectly given as ‘Women’s Knowledge and Health Care-Seeking Behavior Regarding Pelvic Organ Prolapse in the West Bank, Palestine in maternaity and obstatric of Nursing’ but should have been ‘Women’s Knowledge and Health Care-Seeking Behavior Regarding Pelvic Organ Prolapse in the West Bank, Palestine’. 

The original article has been corrected.
