# Supplementary material for: Women’s Knowledge and Health Care-Seeking Behavior Regarding Pelvic Organ Prolapse in the West Bank, Palestine
Source: BMC Womens Health. 2025 Oct 31;25:529. doi: 10.1186/s12905-025-04023-4 (PMC12577352; doi:10.1186/s12905-025-04023-4)
Supplement: Supplementary file 1 — Supplementary Material 1. [file 12905_2025_4023_MOESM1_ESM.docx]

**جامعة القدس/ ابوديس**

**معرفة المرأة الفلسطينية بهبوط أعضاء الحوض** **والعوامل التي تؤثر في طلب الرعاية الصحية في جنوب الضفة الغربية ، فلسطين**

**عزيزتي المشاركة:**

**تحية طيبة وبعد،**

تهدف هذه الدراسة إلى تقييم معرفة المرأة الفلسطينية بهبوط أعضاء الحوض ونسبة انتشار أعراضه والعوامل التي تؤثر على طلب الرعاية الصحية بين النساء الفلسطينيات في جنوب الضفة الغربية، فلسطين. **يعرف هبوط أعضاء الحوض / التهبيطة بأنه هبوط أو تدلي الأعضاء داخل القناة المهبلية أو فتحة الشرج أو خارجها، بما في ذلك: المثانة والرحم والمهبل والمستقيم.**

**عزيزتي المشاركة:**

في حال موافقتك على المشاركة في هذه الدراسة، فان مشاركتُكِ طوعيّة وبإمكانك الانسحاب باي وقت من الدراسة، وتتطلب مشاركتك تعبئة الاستبيان الحالي، وتحتاج تعبئة هذا الاستبيان من خمسة الى عشرة دقائق تقريباً. إنّ المعلومات التي ستزوديننا بها مفيدة جداً لتحقيق الهدف الأساسي من هذه الدراسة، لذا، يرجى تحرّي الدّقة والمصداقيّة في الإجابة عن الأسئلة، لتعكس حقيقة رأيُكِ بشكل سليم، وسيكون رأيُكِ محل التقدير والاعتبار، وسوف تنتهي مشاركتُكِ في البحث بمجرد الانتهاء من الإجابة على الاستبيان.

جميع المعلومات التي سيتم جمعها خلال البحث سيتم التعامل معها بسرية تامّة من قبل الباحثة، إذ أنّه سيتم جمع المعلومات دون التطرُّق لهوية المشارِكة، ولا لأيّ معلومة قد تدل على هويتها.

في حال لديك اي استفسار يمكنك التواصل مع الباحثة الرئيسة

طالبة الماجستير: ضحى محيسن

**بإشراف د. ابتسام دويكات**

**شكرا لمشاركتُكِ في تعبئة هذا الاستبيان**

**القسم الاول:**

**البيانات الديموغرافية**

| **العمر** | | | | | | | |
| --- | --- | --- | --- | --- | --- | --- | --- |
| **أقل من 30 سنة** | | **من 30 – 40 سنة** | | | | **أكثر من 40 سنة** | |
| **الوزن ------------------------ الطول---------------------** | | | | | | | |
| **مستوى التعليم:** | | | | | | | |
| أساسي | ثانوي | | جامعي | | غير ذلك | | |
| **في حال اكملت دراستك الجامعية هل تخصصك الجامعي له علاقة بالمجال الطبي (تمريض، طب بشري…(** | | | | | | | |
| نعم | لا | | لم أكمل تعليمي الجامعي | | | | |
| **العمل:** | | | | | | | |
| ربة منزل | وظيفة مكتبية | | | | تتطلب الوظيفة أنشطة بدنية شاقة مثل رفع الأثقال... | | |
| **مكان الإقامة:** | | | | | | | |
| مدينة | قرية | | | مخيم | | | غير ذلك |
| **الحالة الاجتماعية:**   \| عزباء \| متزوجة \| \| مطلقة \| أرملة \| \| --- \| --- \| --- \| --- \| --- \| \| **ما هو الدخل الشهري المقدر لعائلتكِ؟** \| \| \| \| \| \| \| أقل من 3500 \| \| 3500 او أكثر \| \| \| \| | | | | | | | |
| **ما هو مصدر معلوماتك عن تدلي أعضاء الحوض/ التهبيطة؟ (ممكن الاجابة بأكثر من خيار)** | | | | | | | |
| وسائل التواصل الاجتماعي | | | | الجامعة | | | |
| مقدمو الرعاية الصحية (أطباء، قابلات...) | | | | لم أسمع عن تدلي أعضاء الحوض/ التهبيطة. | | | |
| المجتمع المحيط (زميلات العمل،الأقارب...). | | | | غير ذلك | | | |

**تاريخ الدورة الشهرية**

| **تاريخ الدورة الشهرية** | **نعم** | **لا** |
| --- | --- | --- |
| منتظمة |  |  |
| انقطعت منذ أكثر من عام (سن الامان) |  |  |

**تاريخ الحمولات والولادات:**

|  | **لا يوجد** | **ولادة واحدة** | **2-3 ولادات** | **4 فما فوق** |
| --- | --- | --- | --- | --- |
| عدد الحمولات بما في ذلك التنزيل |  |  |  |  |
| عدد الولادات |  |  |  |  |
| عدد الولادات الطبيعية |  |  |  |  |
| عدد الولادات باستخدام الشفط او ملاقط |  |  |  |  |
| عدد الولادات بعملية قيصرية |  |  |  |  |

|  | لا يوجد ولادات | نعم (يوجد ولادات أكثر من 4 كغم) | لا (لا يوجد ولادات أكثر من 4 كغم) |
| --- | --- | --- | --- |
| هل يوجد ولادات أوزانها أكثر من 4 كغم؟ |  |  |  |

**التاريخ الطبي:**

| **التاريخ الطبي:** | **نعم** | **لا** |
| --- | --- | --- |
| سلس البول (فقدان القدرة على التحكم في المثانة، وهي تسريب البول بين الحين والآخر مع العطس او السعال او.....) |  |  |
| تم اجراء جراحة سابقة لمعالجة سلس البول |  |  |
| تم اجراء جراحة سابقة لمعالجة تدلي أعضاء الحوض (التهبيطة) |  |  |
| تاريخ مرضي مزمن في الصدر (/ سعال مزمن) |  |  |
| الإمساك المزمن |  |  |
| السكري |  |  |
| التدخين (السجائر او ارجيلة) |  |  |
| سرطان بمنطقة الحوض مثل: سرطان الرحم، المبايض .... |  |  |

**القسم الثاني: درجة المعرفة** **حول هبوط أعضاء الحوض ( التهبيطة)**

| **البند** | **لا** | **نعم** | **لا أعلم** |
| --- | --- | --- | --- |
| **معرفتكِ فيما يتعلق بعوامل الخطر (الأسباب التي قد تؤدي) لهبوط أعضاء الحوض (التهبيطة):** | | | |
| يعتبر هبوط أعضاء الحوض أكثر شيوعًا عند النساء الصغار بالعمر |  |  |  |
| قد تؤدي تعدد الولادات إلى هبوط أعضاء الحوض |  |  |  |
| يمكن أن يحدث هبوط أعضاء الحوض في أي عمر |  |  |  |
| يمكن أن يؤدي رفع الأشياء الثقيلة بشكل يومي إلى حدوث هبوط في أعضاء الحوض. |  |  |  |
| من المرجح أن تصاب السيدات المسنات بهبوط أعضاء الحوض. |  |  |  |
| الوزن الزائد يمكن ان يؤدي لحدوث هبوط أعضاء الحوض (التهبيطة) |  |  |  |
| ممكن ان تلعب الوراثة دور بحدوث هبوط أعضاء الحوض. |  |  |  |
| **معرفتكِ فيما يتعلق بتشخيص هبوط أعضاء الحوض (التهبيطة):** | | | |
| الفحص السريري الذي يجريه الطبيب للسيدة هو طريقة جيدة لتشخيص هبوط أعضاء الحوض. |  |  |  |
| فحص الدم يمكن ان يشخص هبوط أعضاء الحوض. |  |  |  |
| يمكن استخدام الرنين المغناطيسي والتصوير الطبقي لتشخيص تدلي أعضاء الحوض. |  |  |  |
| **معرفتكِ فيما يتعلق بعلاج هبوط أعضاء الرحم (التهبيطة):** | | | |
| بمجرد حدوث هبوط في أعضاء الحوض لدى السيدة، لا يمكن فعل الكثير لمساعدتها. |  |  |  |
| في المراحل الأولى من هبوط أعضاء الحوض، يمكن بتعديل نمط الحياة مثل تخفيف الوزن في حالة السمنة والتوقف عن التدخين بتقليل وربما معالجة الاعراض. |  |  |  |
| يمكن أن تساعد بعض التمارين في منع تدهور حالة هبوط أعضاء الحوض. |  |  |  |
| يوجد ادوية تساعد على علاج هبوط أعضاء الحوض. |  |  |  |
| الجراحة هي خيار مطروح لعلاج هبوط أعضاء الحوض. |  |  |  |
| يمكن استخدام حلقة مطاطية تسمى pessary ، لعلاج أعراض هبوط أعضاء الحوض. |  |  |  |
| استئصال الرحم هو التصحيح الوحيد الممكن لهبوط أعضاء الحوض. |  |  |  |

**اعراض هبوط أعضاء الحوض ( التهبيطة)**

يرجى الاجابة عن الاسئلة التالية

| **لا ابدا** | **أحيانا** | **معظم الوقت** | **دائما** | **الاعراض** |
| --- | --- | --- | --- | --- |
|  |  |  |  | هل تعانين من ضغط في أسفل البطن؟ |
|  |  |  |  | هل تعانين من ثقل أو كسل في منطقة الحوض؟ |
|  |  |  |  | هل تشعرين بانتفاخ اووجود كتلة يمكنك رؤيتها أو الشعور بها في منطقة المهبل وخارجه؟ |
|  |  |  |  | هل تحتاجين إلى ادخال او الضغط بإصبعك في منطقة المهبل من اجل التبول الكامل؟ |
|  |  |  |  | هل تعانين من الشعور بعدم اكتمال إفراغ المثانة؟ |
|  |  |  |  | هل تحتاجين إلى الضغط على المهبل أو حول المستقيم للتبرز(الاخراج) ؟ |

| **في حال أنكِ عانيتِ من هبوط أعضاء الحوض او أحد الاعراض المذكورة سابقا، هل لجأت لطلب الرعاية الصحية بهذا الخصوص فور ظهور هذه الأعراض؟** |
| --- |
| نعم لا 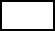 لم أعاني من الاعراض المذكورة سابقا  ان كان الجواب لا، من فضلك أجيبي عن الاسئلة في **القسم الرابع**:  **القسم الرابع:**  **العوامل التي منعتك من طلب المشورة الطبية بالنسبة لهبوط أعضاء الحوض** |

| **العوامل التي منعتك من طلب المشورة الطبية بالنسبة لهبوط أعضاء الحوض** | **نعم** | **لا** |
| --- | --- | --- |
| لأن الاعراض التي تعانين منها غير مزعجة في البداية |  |  |
| عدم المعرفة بأن هذه المشكلة هي هبوط في أعضاء الحوض |  |  |
| الاعتقاد بأن الحالة التي تعانيها هي أمر طبيعي |  |  |
| عدم المعرفة بوجود علاج طبي للمشكلة |  |  |
| الخوف من تأثير اخذ العلاج على عملية الحمل في المستقبل |  |  |
| الشعور بالإحراج من الحديث عن هذه المشكلة للطبيب |  |  |
| الشعور بالإحراج من الفحوصات الطبية التي قد تجرى |  |  |
| عدم القدرة على دفع تكلفة العلاجات الطبية |  |  |
| اسباب مجتمعية – ثقافية |  |  |
| الخوف من ان يكون العلاج بالعمليات الجراحية |  |  |

**شكرا على تعاونك**
