# Supplementary material for: Women’s Knowledge and Health Care-Seeking Behavior Regarding Pelvic Organ Prolapse in the West Bank, Palestine
Source: BMC Womens Health. 2025 Oct 31;25:529. doi: 10.1186/s12905-025-04023-4 (PMC12577352; doi:10.1186/s12905-025-04023-4)
Supplement: Supplementary file 2 — Supplementary Material 2. [file 12905_2025_4023_MOESM2_ESM.docx]

**Al-Quds University**

**Women’s Knowledge and Health Care Seeking Behaviors about Pelvic Organ Prolapse in West Bank, Palestine**

**Dear participants:**

**Greetings,**

This study aims to evaluate Palestinian women's knowledge of pelvic organ prolapse, the prevalence of its symptoms, and the factors that affect health care seeking among Palestinian women in the southern West Bank, Palestine.

**Dear participants:**

If you agree to participate in this study, your participation is voluntary and you can withdraw from the study at any time. Your participation requires filling out the current questionnaire, and filling out this questionnaire takes approximately five to ten minutes. The information that you will provide us is very useful for achieving the primary goal of this study. Therefore, please be accurate and credible in answering the questions, to reflect the truth of your opinion correctly. Your opinion will be appreciated and taken into account, and your participation in the research will end once you have completed answering the questionnaire.

All information that will be collected during the research will be treated with complete confidentiality, as the information will be collected without mentioning the identity of the participant, nor any information that may indicate her identity.

If you have any questions, you can contact the main researcher:

- Master’s student: Doha Moheasen
- Supervised by Dr. Ibtisam Dwekat

**Section one:**

**Demographic data**

| Age | | | | | | | | |
| --- | --- | --- | --- | --- | --- | --- | --- | --- |
| --- less than 30 years | | | --- 30 to 40 years | | | --- more than 40 years | | |
| Weight ------ | | | | | Hight ------ | | | |
| Educational level: | | | | | | | | |
| --- Basic education | --- High school | | | | --- College or university | | | --- Other |
| University major related to medical specializations | | | | | | | | |
| --- Yes | | | | --- No | | | | |
| Occupation: | | | | | | | | |
| --- Housewives | | --- Office job | | | | | --- Jobs require strenuous physical activities | |
| Residency: | | | | | | | | |
| --- City | --- Village | | | | --- Camp | | | --- remote area |
| marital status: | | | | | | | | |
| --- Single | --- married | | | | --- divorced | | | --- widow |
| Monthly family income: | | | | | | | | |
| --- less than 3500 NIC | | | | --- more than 3500 NIC | | | | |
| Source of knowledge about POP and awareness: | | | | | | | | |
| --- Social media | | | | --- Health care provider | | | | |
| --- Surrounding community | | | | --- Through university study | | | | |
| --- I did not hear about POP | | | | --- Heard about POP from other sources | | | | |

**Menstrual history:**

| Menstrual history: | Yes | No |
| --- | --- | --- |
| Regular |  |  |
| Reach menopausal period |  |  |

**Obstetrical history:**

|  | Nothing | One birth | 2 -3 births | More than 4 births |
| --- | --- | --- | --- | --- |
| Number of pregnancies |  |  |  |  |
| Number of births |  |  |  |  |
| Number of normal deliveries |  |  |  |  |
| Number of cesarian section deliveries |  |  |  |  |
| Number of instrumental deliveries |  |  |  |  |

| Are there any births weighing more than 4 kg? | No births | Yes (there are births weighing more than 4 kg) | No (no deliveries over 4 kg) |
| --- | --- | --- | --- |
|  |  |  |  |

**Medical and gynecological history:**

| **Medical and gynecological history** | **Yes** | **No** |
| --- | --- | --- |
| Stress incontinent |  |  |
| Previous surgery for stress incontinent |  |  |
| Previous surgery for POP |  |  |
| Chronic chest illness |  |  |
| Constipation |  |  |
| Diabetes |  |  |
| Cigarette or Nargileh smoking |  |  |
| Cancer in the Pelvic area |  |  |

**Section two:**

**Degree of knowledge about pelvic organ prolapse:**

| Items | Yes | No | I don’t know |
| --- | --- | --- | --- |
| **Knowledge regarding risk factors for pelvic organ prolapse** | | | |
| Pelvic organ prolapse is more common in young women. |  |  |  |
| Multiple births may lead to pelvic organ prolapse |  |  |  |
| Pelvic organ prolapse can occur at any age |  |  |  |
| Lifting heavy objects daily can lead to pelvic organ prolapse |  |  |  |
| Elderly women are more likely to suffer from pelvic organ prolapse |  |  |  |
| Overweight can lead to pelvic organ prolapse |  |  |  |
| Genetics may play a role in the development of pelvic organs |  |  |  |
| **Knowledge regarding the diagnosis of pelvic organ prolapse** | | | |
| A clinical examination conducted by a doctor is a good way to diagnose pelvic organ prolapse |  |  |  |
| A blood test can diagnose pelvic organ prolapse |  |  |  |
| Magnetic resonance imaging (MRI) and computed tomography (CT) can be used to diagnose pelvic organ prolapse. |  |  |  |
| **Knowledge regarding the treatment of uterine prolapse** | | | |
| Once a woman has prolapsed pelvic organs, not much can be done to help her. |  |  |  |
| In the early stages of pelvic organ prolapse, lifestyle modifications, such as losing weight in the case of obesity and stopping smoking, can reduce and possibly treat the symptoms. |  |  |  |
| Certain exercises “Kegel exercise” can help prevent pelvic organ prolapse from getting worse. |  |  |  |
| There are medications help treat pelvic organ prolapse. |  |  |  |
| Surgery is an option for treating pelvic organ prolapse. |  |  |  |
| A rubber ring, called a pessary, may be used to treat symptoms of pelvic organ prolapse. |  |  |  |
| Hysterectomy is the only possible correction of pelvic organ prolapse. |  |  |  |

**Section three:**

**Symptoms of organ prolapse:**

Please answer the following questions

| **Symptoms** | **Never** | **Sometimes** | **Often** | **Always** |
| --- | --- | --- | --- | --- |
| Do you suffer from lower abdominal pressure? |  |  |  |  |
| Do you suffer from heaviness in the pelvic area? |  |  |  |  |
| Do you have a bulge or lump that can be seen or felt in or outside the vaginal area? |  |  |  |  |
| Do you need to insert or press your finger into the vaginal area in order to urinate completely? |  |  |  |  |
| Do you suffer from a feeling of incomplete bladder emptying? |  |  |  |  |
| Do you need to apply pressure on the vagina or around the rectum to defecate? |  |  |  |  |

If you suffered from pelvic organ prolapse or one of the symptoms mentioned previously, did you seek health care **immediately after these symptoms appeared**?

| ----- Yes | ----- No | ----- I did not suffer from the symptoms mentioned previously |
| --- | --- | --- |

If the answer is no, please answer the questions in section four:

**Section four:**

**Factors that prevented from seeking medical care for pelvic organ prolapse**

| **Factors that prevented from seeking medical care for pelvic organ prolapse:** | Yes | No |
| --- | --- | --- |
| The symptoms are not annoying at first. |  |  |
| Belief that the condition they suffer is normal. |  |  |
| Lack of knowledge of the existence of a medical treatment for the problem. |  |  |
| Fear that treatment will be limited to surgery. |  |  |
| Feel embarrassed to talk about this problem to the doctor. |  |  |
| Embarrassment from medical assessment that may be performed. |  |  |
| Inability to pay the cost of medical treatment. |  |  |
| Fear of the effect of treatment on pregnancy in the future. |  |  |
| Societal-cultural reasons. |  |  |
| Lack of knowledge that there is a medical treatment for a problem |  |  |

**Thank you**
